# Quantum Bounds on the Generalized Lyapunov Exponents

**DOI:** 10.3390/e25020246

**Published:** 2023-01-30

**Authors:** Silvia Pappalardi, Jorge Kurchan

**Affiliations:** Laboratoire de Physique de l’École Normale Supérieure, ENS, Université PSL, CNRS, Sorbonne Université, Université de Paris, F-75005 Paris, France

**Keywords:** quantum chaos, generalized Lyapunov exponents, quantum bound to chaos

## Abstract

We discuss the generalized quantum Lyapunov exponents $L_{q}$, defined from the growth rate of the powers of the square commutator. They may be related to an appropriately defined thermodynamic limit of the spectrum of the commutator, which plays the role of a large deviation function, obtained from the exponents $L_{q}$ via a Legendre transform. We show that such exponents obey a generalized bound to chaos due to the fluctuation–dissipation theorem, as already discussed in the literature. The bounds for larger *q* are actually stronger, placing a limit on the large deviations of chaotic properties. Our findings at infinite temperature are exemplified by a numerical study of the kicked top, a paradigmatic model of quantum chaos.

## 1. Introduction

Classical chaos is well understood from the sensitivity of the dynamics to respect small changes in the initial conditions, the so-called butterfly effect. This is quantified by the Lyapunov exponent, the rate at which nearby trajectories separate exponentially in time. In the past few years, there has been a lot of attention given to quantum chaos and in particular, on the *quantum Lyapunov exponent* $\lambda_{L}$, defined from the intermediate-time exponential growth of the following square-commutator:

(1) $$\langle {\left|[A(t),B]\right|}^{2}\rangle ∼\epsilon^{2}e^{\lambda_{L}t}\;,$$

where ϵ is a small parameter [1]. The interest in this object comes from the fact that $\lambda_{L}$ obeys a bound:

(2) $$\lambda_{L}\leq \frac{2\pi }{\beta \hbar }\;.$$

This result, now known as the *quantum bound to chaos*, was proved within the high energy community [2], driven to the topic because maximal chaos is attained by models of black holes, including the Sachdev–Ye–Kitaev model (SYK) [3,4,5]. See also Refs. [6,7] for alternative derivations. The interest in these issues has later spread over different communities, from condensed matter to quantum information theory. Recently, the existence of the bound in Equation (2) was physically rationalized as a consequence of the fluctuation–dissipation theorem (FDT) [8,9] since the out of time-order correlators (OTOC) appearing in Equation (1) can be mapped into two-time correlation functions in a duplicated Hilbert space.

Actually, both classically and quantum mechanically, the Lyapunov exponent is a distributed quantity: different starting conditions—or different time intervals after the same starting condition—yield different exponents, and they are peaked on a ‘typical’ value and have large (and rare) deviations around, for example, when a classical trajectory grazes a regular region. If the distribution is not a delta function, it is referred to as ‘multifractal’ [10].

A possibility to study the full distribution of Lyapunov exponents is to introduce the generalized Lyapunov exponents (GLE) $L_{2q}$, defined from the moments of the distribution (This may lead to confusion. The Lyapunov exponents are classified as *first (maximal)*, *second*, … according to whether they measure linear, area, and in general *k*-form expansions [11]. Each is also generalized according to the moment *q* considered. Here we are considering expansions of linear lengths, and all moments *q* of the ‘first, maximum, Lyapunov exponent’, thus $L_{2q}^{\left(k\right)}$ for all *q* and k=1). It turns out that the *quantum* generalized exponents also satisfy themselves a bound, stated in Ref. [8], that generalizes Equation (2). These bounds are the subject of this paper. We have two motivations: Firstly, they put limitations on the chaotic properties of *rare* protocols, favoring high and low chaoticity, and also allow us to define a Lyapunov exponent for *typical* protocols, actually different from the usual one considered in quantum mechanics. Secondly, a system that approaches the bounds for all the $L_{2q}$ will turn out to be *mono-fractal*, i.e, the large deviation function becomes peaked on a single value, a property we find intriguing.

The quantum generalized Lyapunov exponents are defined by considering the 2q-th commutator between two operators at different times, and assuming that they scale exponentially with time as

(3) $$G_{2q}\left(t\right)={\langle {\left(i[A(t),B)]\right)}^{2q}\rangle }_{\beta }∼\epsilon^{2q}\;e^{L_{2q}^{\left(\beta \right)}t}\;,$$

where ϵ is a small parameter, two common examples are ϵ=ℏ (the semiclassical limit) or $\epsilon =N^{-1}$ (as in the SYK model), the latter being more relevant here as we shall be interested in thermodynamic models. The thermal average ${\langle •\rangle }_{\beta }$ at inverse temperature β may be defined in various ways, as we shall see below.

The rate $L_{2q}^{\left(\beta \right)}$ now defines the *quantum thermal* generalized Lyapunov exponent of order *q*. The usual (quenched) Lyapunov exponent thus is

(4) $$\begin{array}{c} \lambda_{1}^{\left(\beta \right)}=\lim_{q\to 0}\;\frac{L_{2q}^{\left(\beta \right)}}{2q}\; \end{array}$$

On the other hand, the grow rate of the square commutator $\lambda_{L}$ actually corresponds to the GLE with q=1: $\lambda_{L}=L_{2}^{\left(\beta \right)}$.

In the quantum realm, the exponential regime holds only at intermediate times, up to the so-called *Ehrenfest time* $t_{\mathrm{Ehr}}∼\ln\epsilon^{-1}$. In actual fact, only when $\lim t_{\mathrm{Ehr}}\to \infty$ is the Lyapunov regime unambiguously defined.

Different *q*-Lyapunovs are dominated by initial conditions having different expansion rates, with larger rates dominating the averages corresponding to larger *q*. Hence, one must allow for the dependence on the Ehrenfest time itself:

(5) $$t_{\mathrm{Ehr}}^{\left(2q\right)}=\frac{2q}{L_{2q}}\ln\epsilon^{-1}\;\mathrm{such}\;\mathrm{that}\;G_{2q}\left(t\right)∼e^{L_{2q}^{\left(\beta \right)}\left(t-t_{\mathrm{Ehr}}^{\left(2q\right)}\right)}\;.$$

A crucial assumption we shall make here is that

(6) $$t_{\mathrm{Ehr}}^{\left(2q\right)}\leq t_{\mathrm{Ehr}}^{\left(2q^{\prime }\right)}\;for\;q>q^{\prime }$$

Under these assumptions, we show that the following holds:

(7) $$\frac{L_{2q}^{\left(\beta \right)}}{2q}\leq \frac{\pi }{\beta \hbar }\;.$$

This bound was already stated in Ref. [8], without the identification of the rate $L_{2q}^{\left(\beta \right)}$ as a quantum GLE and the relation to large deviations. We will also clarify some assumptions on which the derivation [8].

Because we are assuming that $\frac{L_{2q}^{\left(\beta \right)}}{2q}$ does not decrease, the bounds for larger *q* (but always of order one with respect to *N*) are more stringent. The meaning of this, as the classical discussion below will make clear, is that even rarely expanding conditions are bounded.

## 2. Classical Generalized Lyapunov Exponents

Let us briefly review the classical case [11]. Consider the infinitesimal separation |Δ(t)| between two trajectories at time *t*, starting at a point $x_{0}$ and $x_{0}+\Delta$:

(8) $$R\left(t\right)=\frac{\left|\Delta (t)\right|}{\left|\Delta (0)\right|}∼e^{\lambda \;t}\;.$$

The rate λ is a function of the initial condition $x_{0}$. This quantity grows in time according to an asymptotic exponential law λt with (λ>0) if the system is chaotic, where λ is a function of time which reaches a finite limit at long times. A very long chaotic trajectory will have explored most of the phase space, and the exponential expansion will be sampled from all regions: the value λ then becomes essentially the same for all initial conditions. This fact is encompassed in the Oseledec theorem [12].

The fact that lnR(t) is a cumulative process over stretches of time with uncorrelated properties leads to the usual argument for the introduction of a large deviation principle, in this case for the probability of a Lyapunov value given a random initial condition:

(9) lnP(λ,t)∼−tS(λ),

where *S* is the large-deviation (Cramér) function. If the system is ergodic, the ensemble of initial conditions may be substituted by the ensemble of initial times along the same trajectory.

The typical Lyapunov exponent $\lambda_{1}$ is given by

(10) $$\lambda_{1}\equiv \lim_{t\to \infty }\frac{1}{t}{\langle \ln R\left(t\right)\rangle }_{\tau }\equiv \lim_{t\to \infty }\int d\lambda \;P\left(\lambda ,t\right)\lambda$$

(note that λ=λ(t)).

One can study the 2q-th moments which, for long enough times, shall grow exponentially as

(11) $$R_{2q}\left(t\right)=\langle R{\left(t\right)}^{2q}\rangle ∼e^{L_{2q}t},$$

where

(12) $$L_{2q}=\lim_{t\to \infty }\frac{1}{t}\ln\langle R{\left(\tau \right)}^{2q}\rangle$$

are called the generalized Lyapunov exponents (GLE) of order 2q, and characterize the fluctuations of the dynamical system [10]; see also Ref. [13]. They are defined as *annealed averages*:

(13) $$L_{2q}\equiv \frac{1}{t}\ln\lim_{t\to \infty }\int d\lambda \;P\left(\lambda ,t\right)\;e^{2q\lambda t}∼\max_{\lambda }\left\{-S(\lambda )+2q\lambda \right\}\;,$$

where we have evaluated the integral over large *t* using saddle point. The typical Lyapunov exponent is retrieved as the limit

(14) $$\lambda_{1}=\lim_{q\to 0}\frac{L_{2q}}{2q}=\frac{1}{2}\frac{dL_{2q}}{dq}{|}_{q=0}\;.$$

The GLE $L_{2q}$ is the Legendre transform of S(λ), via Equation (13). As such, also $L_{2q}$ is a convex function of 2q. As a consequence, one has that $L_{2q}$ obeys two important properties:

1. $L_{2q}/2q$ are an increasing function of the order *q*:
  (15) $$\frac{d}{dq}\left(\frac{L_{2q}}{2q}\right)\geq 0\;;$$
2. The GLEs are always bounded by the linear behavior:
  (16) $$L_{2q}\geq 2q\lambda_{1}\;.$$

These equations are obtained via the property of convex differentiable functions f(x): $f\left(x\right)\geq f\left(y\right)+f^{\prime }\left(y\right)\left(x-y\right)$. For x=2q and y=0, this yields Equation (16), while for x=0 and y=2q, one has Equation (15).

The equality $L_{2q}=2q\lambda_{1}$ in Equation (16) holds only if $P\left(\lambda \right)=\delta \left(\lambda -\lambda_{1}\right)$, namely if the Lyapunov exponent is the same and does not fluctuate, we have *mono-fractality*. Interestingly, this characterizes also random matrices of dimension *D* without any structure and with high connectivity, which satisfy $L_{2q}=2q\lambda_{1}+O\left(1/D\right)$ [10]. Otherwise, the system is characterized by *multifractal behavior*. The higher the moments, the more important the contributions coming from the tales of the distribution. In particular, in the case of a distribution P(λ) with a finite support, the limits

(17) $$\lambda_{max/min}=\lim_{q\to \pm \infty }\frac{L_{2q}}{2q}$$

select the maximal and minimal expanding rates.

## 3. Quantum Generalized Lyapunov Exponents at Infinite Temperature

Our goal is to extend the definition of generalized Lyapunov exponents to the quantum domain to discuss the bound in Equation (7). Systems with a few degrees of freedom do not lend themselves to the implementation of bounds that depend on temperature, as the canonical ensemble is not particularly useful for them. However, we may understand some other features that are also valid in thermodynamic systems by studying infinite-temperature systems of this kind. In this section, we define the quantum generalized Lyapunov exponents at infinite temperature and discuss their “convexity” properties. We will see that it is straightforward to interpret the quantum GLE as a probe of the spectral properties of the square-commutator operator.

### 3.1. Properties of the Infinite Temperature Quantum GLE

Let us first analyze the infinite temperature 2q-th commutator Equation (3) at infinite temperature:

(18) $$G_{2q}^{\left(0\right)}\left(t\right)=\frac{{\left(-1\right)}^{q}}{Z}\;\mathrm{Tr}\left({\left[A\left(t\right),B\right]}^{2q}\right),$$

where Z=Tr(I)=dimH is given by the Hilbert space dimension. This object generalizes the infinite temperature square-commutator in Equation (1), which has been discussed in a variety of models, and it is particularly relevant for dynamical protocols where energy is not conserved (such as with periodic driving or in the open system’s scenario).

The infinite temperature quantum GLEs are then defined by the exponential growth at intermediate times:

(19) $$G_{2q}^{\left(0\right)}\left(t\right)∼\epsilon^{2q}e^{L_{2q}^{\left(0\right)}t}\;.$$

We now show that $L_{2q}^{\left(0\right)}$ obey the same properties as the classical ones (e.g., Equation (15)). Let us re-write Equation (18) as

(20) $$G_{2q}^{\left(0\right)}\left(t\right)=\frac{1}{Z}\sum_{i}{\left(g_{i}\left(t\right)\right)}^{2q}\;,$$

where we defined $g_{i}\left(t\right)$ the eigenvalues of the square-commutator operator, i.e.,

(21) $$-{\left[A\left(t\right),B\right]}^{2}=\sum_{i_{t}}{\left(g_{i}\left(t\right)\right)}^{2}|i_{t}\rangle \langle i_{t}|\;.$$

and we have made explicit a factor *t* so that $\lambda_{t}$ may have a finite limit. Some properties of this operator have been studied on specific models, see, for example, Refs. [14,15]. If the expectation value of the square commutator grows exponentially (we consider only times before $t_{\mathrm{Ehr}}$), then it is convenient to write each eigenvalue as

(22) $$g_{i}\left(t\right)=e^{\lambda_{t}^{i}t}\;.$$

By using $1=\int d\lambda \delta (\lambda -\lambda_{t}^{i})$, we can re-write Equation (18) as

(23) $$G_{2q}^{\left(0\right)}\left(t\right)=\int d\lambda \;P\left(\lambda \right)e^{2q\lambda t}$$

where we have defined the *distribution of the quantum local Lyapunov exponents*

(24) $$P\left(\lambda \right)=\sum_{i}\delta \left(\lambda_{i_{t}}-\lambda \right)\;.$$

Equations (23) and (24) shows that the $G_{2q}^{\left(0\right)}\left(t\right)$ are moments since they can be written as an integral of times the powers of a function times a positive function P(λ). We can associate to the latter a convex Cramér function tS(λ)∼lnP(λ) as in Equation (9), which gives the Legendre transform of $L_{2q}^{\left(0\right)}$. These relations imply the convexity of the quantum GLE at an infinite temperature, which results in the following:

1. $L_{2q}^{\left(0\right)}/2q$ is an increasing function of *q*;
2. The following inequality holds:
  (25) $$L_{2q}^{\left(0\right)}\geq 2q\lambda_{1}^{\left(0\right)}\;,$$
  where $\lambda_{1}^{\left(0\right)}=\lim_{q\to 0}\frac{L_{2q}^{\left(0\right)}}{2q}$.

The equality holds in the absence of fluctuations in the spectrum of the square-commutator operator. Such mono-fractal behavior means that—for the appropriate time’s range—the square-commutator operator is close to a constant times the identity matrix.

### 3.2. A Semi-Classical Example: The Quantum Kicked Top

As an illustrative example, we study a driven model: the quantum kicked top. Since the energy is not conserved, this model is equivalent to a system at infinite temperature. We thus show that $L_{2q}^{\left(0\right)}$ satisfies the properties of convexity and of large deviation theory. (In this section, we denote $L_{2q}^{\left(0\right)}=L_{2q}$, for the sake of clarity.)

The model is described by the time-dependent Hamiltonian:

(26) $$H=\alpha S_{x}+\frac{J}{N}S_{z}^{2}\sum_{n=-\infty }^{\infty }\delta \left(t-n\tau \right)\;,$$

where $S_{x,y,z}=\frac{1}{2}\sum_{i=1}^{N}\sigma_{x,y,z}^{i}$ are collective spin operators. Due to the collective nature of the interactions, for large *N*, the classical limit is approached. One can define an effective Planck constant:

(27) $$\hbar =\frac{1}{S}=\frac{2}{N}$$

that vanishes in the thermodynamic limit. The stroboscopic time-evolution operator (namely, the time-evolution operator over one period) reads as

(28) $$\hat{U}={\hat{U}}_{J}{\hat{U}}_{\alpha }\;\mathrm{with}\;U_{\alpha }=e^{-i\alpha S_{x}}\;,\;{\hat{U}}_{J}=e^{-i\frac{J}{N}S_{z}^{2}}\;.$$

We fix τ=1 and α=π/2. Changing the value of the kicking strength *J*, this model undergoes a transition between a regular regime and a chaotic one [16,17]. The dynamics of the square commutator (1) have been extensively explored [18,19,20,21,22].

We consider the strongly chaotic limit by choosing J=3.5, and we look at the infinite temperature state with ρ=I/dimH, with dimH=N+1. We study the dynamics of the $G_{2q}\left(t\right)$ via exact numerical calculations, specifically via exact diagonalization. We compute the stroboscopic time-evolution of the 2q-th commutator (3) using $\hat{A}=\hat{B}={\hat{S}}^{z}$ at times t=nτ=0,1,2,….

**Figure 1:**
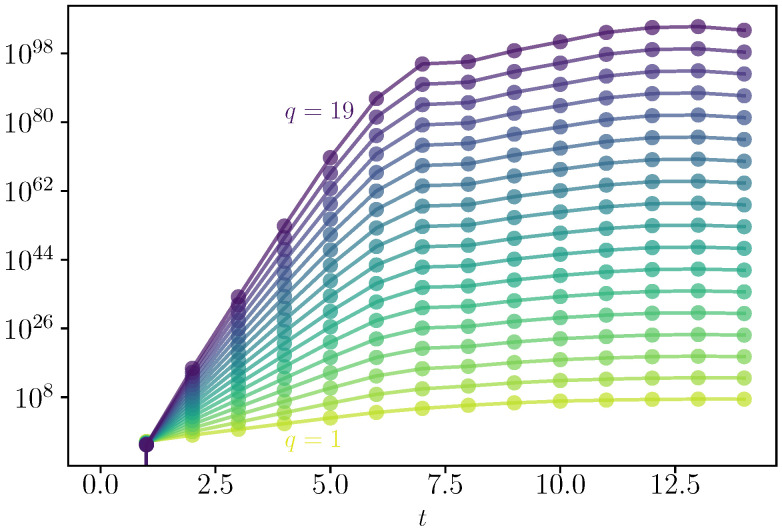
Dynamics of $G_{2q}\left(t\right)$ in Equation (3) for different values of q=1÷19 as a function of time for N=1600.

**Figure 2:**
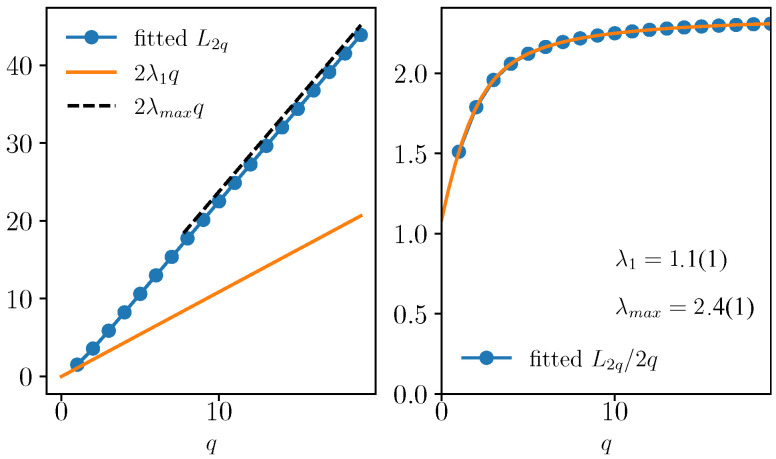
Generalized Lyapunov exponents fitted from Figure 1. (**Left**) $L_{2q}$ as a function of *q*, contrasted with the actual Lyapunov exponent $\lambda_{1}$ and the maximal expanding rate $\lambda_{max}$, obtained by a fit of these data at large *q*. (**Right**) $L_{2q}/2q$ as a function of the moment *q*, from which we extract the maximal Lyapunov exponent $\lambda_{1}$.

In Figure 1, we show the dynamics of $G_{2q}\left(t\right)$ for different values of q=1÷19. The correlators are rescaled by $\hbar^{2q}$ [with ℏ=2/N] to emphasize the scaling of Equation (3). Each commutator grows exponentially before the Ehrenfest times with a different rate that corresponds to the quantum GLE $L_{2q}^{}$. The value of $L_{2q}$ is then fitted and plotted in Figure 2a, where we display its behavior as a function of *q*. It is a convex function of *q* that satisfies $L_{2q}>2q\lambda_{1}$ [cf. Equation (25)], being therefore multifractal. The typical Lyapunov exponent $\lambda_{1}$ is computed as in Figure 2b, where $L_{2q}/2q$ is plotted as a function of *q*. The extrapolation to q→0 yields $\lambda_{1}=1.1\left(1\right)$, which corresponds to the maximum Lyapunov exponent of the classical model in the chaotic phase $\lambda_{class}=1.12$, as computed via the Benettin et al. algorithm [23,24]; see, for example, the appendix of Ref. [22]. We also extract the maximal expanding rate $\lambda_{max}=2.4\left(1\right)$ [cf. Equation (17)] from the limit q→∞, signaling that the distribution of Lyapunov has finite support.

**Figure 3:**
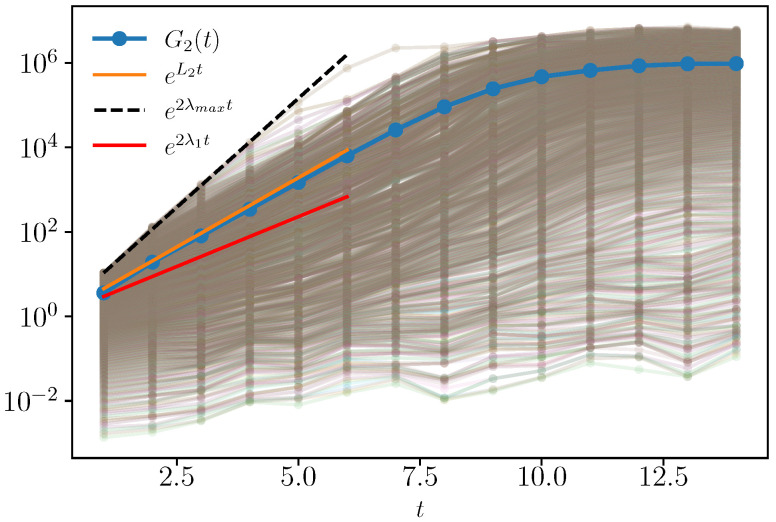
Spectrum of the square commutator $g_{i}^{2}\left(t\right)$, compared with $G_{2}\left(t\right)$ and the exponential growth with $L_{2}$, with the maximal expansion rate $\lambda_{max}$ and with $\lambda_{1}$ for N=1600.

Figure 3 (shaded lines) shows the spectrum of the square commutator in Equation (21). Most of the eigenvalues grow exponentially in time before saturation and thus define some local Lyapunov exponents. We compare this behavior with the standard square-commutator expectation $G_{2}\left(t\right)$ [cf. Equation (1)] (blue dots), which grows exponentially at a rate $L_{2}$ larger than the maximum Lyapunov $\lambda_{1}$. The figure also shows that $\lambda_{max}$ as fitted and extracted in Figure 2 (the dashed black line) corresponds to the maximal expanding rate of the local Lyapunov exponents.

**Figure 4:**
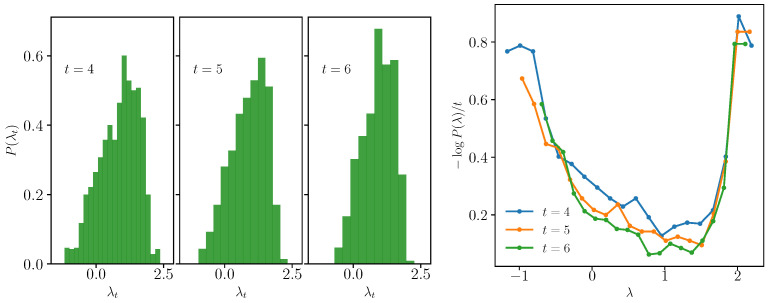
Large deviation properties of the spectrum of the square-commutator for N=1600. (**Left**) Numerical distributions of $\lambda_{t}^{i}=\ln\left(g_{i}\left(t\right)\right)/2t$ with $g_{i}^{2}\left(t\right)$ the eigenvalues of Equation (20) at different times t=3,4,5. (**Right**) −lnP(λ)/t with P(λ) the empirical distribution.

In Figure 4, we show that the local Lyapunov exponents are a large deviation. We consider the coefficients $\lambda_{t}^{i}=\ln\left(g_{i}\right)/2t$ from Figure 3 (we divide everything by a constant factor). On the left, we plot their numerical distribution at different times t=3,4,5, which shows that it converges to a distribution at large *t*. On the right, we plot −lnP(λ)/t, which shall correspond to the smooth convex “Cramer” function at large times; see Equation (9).

## 4. Thermal Quantum Generalized Exponents

It is useful to consider the different *regularizations* of the 2q-th commutator as

(29) $$\begin{array}{cc} G_{2q}^{\left(\beta \right)}\left(t\right) & =\mathrm{Tr}\left({\left(\rho^{\frac{1}{4q}}i\left[A\left(t\right),B\right]\rho^{\frac{1}{4q}}\right)}^{2q}\right)\;,\; \end{array}$$

(30) $$\begin{array}{cc} {\bar{G}}_{2q}^{\left(\beta \right)}\left(t\right) & =\mathrm{Tr}\left({\left(i[\rho^{\frac{1}{8q}}A\left(t\right)\rho^{\frac{1}{8q}},\rho^{\frac{1}{8q}}B\rho^{\frac{1}{8q}}]\right)}^{2q}\right)\;. \end{array}$$

with

(31) $$\rho =e^{-\beta H}/Z_{\beta }\;\mathrm{and}\;Z_{\beta }=\mathrm{Tr}e^{-\beta H}$$

Considering q=1 in Equations (29) and (30), we retrieve the standard regularized square commutators for which the bounds have been proved in Ref. [9]. In Section 4.2 below, we show that the multi-time correlation functions appearing in Equations (29) and (30) can be mapped as two-times functions in a replicated Hilbert space of 2q copies. This allows one to rationalize the use of such regularizations—that might seem an artificial construction—as fixing the temperature of the different replicas to be the same.

These regularizations define the thermal average introduced in Equation (3), which defines thermal GLE $L_{\beta }^{\left(2q\right)}$. We consider the situation in which both of the *q*-th commutators grow exponentially in time as

(32) $$\begin{array}{c} G_{2q}^{\left(\beta \right)}\left(t\right)\propto \epsilon^{2q}e^{L_{2q}^{\left(\beta \right)}t}\;, \end{array}$$

(33) $$\begin{array}{c} {\bar{G}}_{2q}^{\left(\beta \right)}\left(t\right)\propto \epsilon^{2q}e^{L_{2q}^{\left(\beta \right)}t}\;, \end{array}$$

which is valid only for an intermediate time regime

(34) $$t_{d}\ll t\leq t_{\mathrm{Ehr}}^{\left(2q\right)}\equiv \frac{L_{2q}^{\left(\beta \right)}}{2q}\ln\epsilon^{-1}\;.$$

### 4.1. From Commutators to OTOCS

Consider the quantities of Equations (29) and (30). Expanding the commutators, we get a series of OTOC terms containing exactly *k* times:

(35) $$G_{2q}^{\left(\beta \right)}\left(t\right)=\sum_{k=1}^{2q}d_{k}\;{\left[OTOC\right]}_{k}$$

of the form

(36) $${\left[OTOC\right]}_{k}={\langle A_{1}\left(t\right)B_{1}\left(0\right)\ldots A_{k}\left(t\right)B_{k}\left(0\right)\rangle }_{\beta }+h.c.\;,$$

where $A_{1},B_{1},\ldots$ are powers of *A* and *B* respectively and $d_{k}$ some coefficients. With these notations, ${\left[OTOC\right]}_{1}={\langle A^{q}\left(t\right)B^{q}\left(0\right)\rangle }_{\beta }+h.c.$ is a function of two times. Out-of-time-order correlators between *k* operators are sometimes referred to as *k*-OTOC; see Refs. [25,26,27,28]. We are here interested in understanding their structure in time for exponential growth. Following [2], we assume that there exists some dissipation time $t_{d}$, after which two-point functions factorize as ${\left[OTOC\right]}_{1}∼C_{q}$.

Each ${\left[OTOC\right]}_{2k}$ may grow at most as fast as the corresponding Lyapunov behavior, during the corresponding Ehrenfest time:

(37) $$C_{k}-{\left[OTOC\right]}_{2k}\propto \epsilon^{2k}e^{L_{2k}t}=e^{L_{2k}\left(t-t_{\mathrm{Ehr}}^{\left(2k\right)}\right)}\;.$$

If we evaluate this term at times corresponding to a finite but small fraction of the corresponding Ehrenfest time $t_{\mathrm{Ehr}}^{\left(2q\right)}$, we conclude that all the terms with k<q are of lower or equal order, because of the ordering of Ehrenfest times [cf. Equation (6)]. We thus conclude that

(38) G2q(β)(t)∼Cq−ReTrρ12qA(t)Bρ12qA(t)B…ρ12qA(t)B,

(39) $$\begin{array}{cc} {\bar{G}}_{2q}^{\left(\beta \right)}\left(t\right) & ∼{\bar{C}}_{q}-\mathrm{Tr}\left(\;\rho^{\frac{1}{4q}}A\left(t\right)\rho^{\frac{1}{4q}}B\;\rho^{\frac{1}{4q}}A\left(t\right)\rho^{\frac{1}{4q}}B\;\ldots \rho^{\frac{1}{4q}}A\left(t\right)\rho^{\frac{1}{4q}}B\;\right)\;, \end{array}$$

where the constants $C_{q},{\bar{C}}_{q}$ are different given the different regularizations.

### 4.2. Product Space, Fluctuation-Dissipation Theorem and Bound

In this section, we will show how the multi-time OTOC appearing in the generalized 2q-th commutators has a simple interpretation as two-time correlation functions in a 2q-replicated space, see [25]. Focusing on q=1, in Ref. [9] we have stressed that bringing an OTOC into this representation for finite β allows one to write the corresponding fluctuation-dissipation (FDT) relations as a usual KMS one. Here, we will demonstrate it for generic *q*.

Let us consider the following 4q point out of time order correlator:

(40) $$\begin{array}{cc} S_{2q}\left(t\right) & ={\frac{1}{Z}}_{\beta }\mathrm{Tr}\left(\;{\left(\rho^{\frac{1}{2q}}A\left(t\right)B\;\right)}^{2q}\;\right)=\mathrm{Tr}\left(\;\rho^{\frac{1}{2q}}A\left(t\right)B\;\rho^{\frac{1}{2q}}A\left(t\right)B\;\ldots \rho^{\frac{1}{2q}}A\left(t\right)B\;\right)\;. \end{array}$$

Now, we re-write it in terms of the spectral representation of the Hamiltonian $H|n\rangle =E_{n}|n\rangle$ as

$$\begin{array}{cc} S_{2q}\left(t\right) & ={\frac{1}{Z}}_{\beta }\;\sum_{n_{1}n_{2}\ldots n_{2q}}e^{-\frac{\beta }{2q}\left(E_{n_{1}}+E_{n_{2}}+\ldots E_{n_{2q}}\right)}\;\times \langle n_{1}\left|A\left(t\right)B\right|n_{2}\rangle \langle n_{2}\left|A\left(t\right)B\right|n_{3}\rangle \ldots \langle n_{2q}\left|A\left(t\right)B\right|n_{1}\rangle \\ \; & ={\frac{1}{Z}}_{\beta }\sum_{n_{1}n_{2}\ldots n_{2q}}e^{-\frac{\beta }{2q}\left(E_{n_{1}}+E_{n_{2}}+\ldots E_{n_{2q}}\right)}\;\times \langle n_{1}n_{2}\ldots n_{2q-1}n_{2q}\left|A\left(t\right)B\right|n_{2}n_{3}\ldots n_{2q}n_{1}\rangle \;, \end{array}$$

where we introduced the operators that act in the 2q-th replicated Hilbert space:

(41) $$\begin{array}{cc} A(t) & =A(t)⊗A(t)\ldots ⊗A(t)\;,\;B=B⊗B\ldots ⊗B\;, \end{array}$$

and the replicated Hamiltonian

(42) H=H⊗1…⊗1+1⊗H…⊗1+…+1⊗1…⊗H.

We also define the *cyclic shift operator* P that permutes cyclically states between the Hilbert spaces as

(43) $$\begin{array}{cc} P|n_{1}n_{2}\ldots n_{2q-1}n_{2q}\rangle & =|n_{2}n_{3}\ldots n_{q}n_{1}\rangle \;\mathrm{with}\;\;P^{2q}=1\;, \end{array}$$

(44) $$\begin{array}{cc} P^{†}|n_{1}n_{2}\ldots n_{2q-1}n_{2q}\rangle & =|n_{2q}n_{1}n_{2}\ldots n_{2q-1}\rangle \;.\; \end{array}$$

Notice that the operator P is non-Hermitian, but we can define $\tilde{P}=\frac{P+P^{†}}{2}$ that is. $\tilde{P}$ also commutes with A(t), B and H so that $\tilde{P}B$ is Hermitian.

Let us re-write Equation (41) as

$$\begin{array}{cc} S_{2q}\left(t\right) & ={\frac{1}{Z}}_{\beta }\sum_{n_{1}n_{2}\ldots n_{2q}}e^{-\frac{\beta }{2q}\left(E_{n_{1}}+E_{n_{2}}+\ldots E_{n_{2q}}\right)}\times \frac{1}{2}(\langle n_{1}n_{2}\ldots n_{2q-1}n_{2q}\left|A\left(t\right)B\right|n_{2}n_{3}\ldots n_{q}n_{1}\rangle \\ & \;+\langle n_{1}n_{2}\ldots n_{2q-1}n_{2q}|A\left(t\right)B|n_{2q}n_{1}\ldots n_{q-2}n_{2q-1}\rangle )\;, \end{array}$$

where in the second line, we simply used a different resolution of the identity and a reshuffling of the matrix elements (We use

$$\begin{array}{cc} \; & \langle n_{1}\left|A\left(t\right)B\right|n_{2q}\rangle \langle n_{2q}\left|A\left(t\right)B\right|n_{2q-1}\rangle \ldots \langle n_{3}\left|A\left(t\right)B\right|n_{2}\rangle \langle n_{2}\left|A\left(t\right)B\right|n_{1}\rangle =\\ & \;\;\langle n_{1}\left|A\left(t\right)B\right|n_{2q}\rangle \langle n_{2}\left|A\left(t\right)B\right|n_{1}\rangle \langle n_{3}\left|A\left(t\right)B\right|n_{2}\rangle \ldots \langle n_{2q}\left|A\left(t\right)B\right|n_{2q-1}\rangle \;. \end{array}$$).

Therefore, we can re-write Equation (40) as

(45) $$S_{2q}\left(t\right)={\frac{1}{Z}}_{\beta }\mathrm{Tr}\left(e^{-\beta_{2q}H}\;A\left(t\right)B\;\tilde{P}\right)$$

which, besides a normalization, is a standard equilibrium expectation value of a two-time function at inverse temperature $\beta_{2q}=\beta /2q$. This result naturally generalizes the one for four times OTOC, for which $P=P^{†}=\tilde{P}$, as derived in Ref. [9].

#### 4.2.1. Fluctuation–Dissipation in the Replicated Space

We may now write the extended KMS relations. We consider

(46) $$\begin{array}{cc} C_{2q}\left(t\right)= & \frac{1}{2}\frac{1}{Z}\mathrm{Tr}\left[e^{-\beta_{2q}H}\;\left\{A\left(t\right),\;B\;\tilde{P}\right\}\right]\;,\; \end{array}$$

(47) $$\begin{array}{cc} R_{2q}\left(t\right)= & \frac{i}{\hbar }\theta \left(t\right)\frac{1}{Z}\mathrm{Tr}\left[e^{-\beta_{2q}H}\;\left[A\left(t\right),\;B\;\tilde{P}\right]\right] \end{array}$$

(48) $$\begin{array}{cc} F_{2q}\left(t\right)= & \frac{1}{Z}\mathrm{Tr}\left[e^{-\frac{\beta_{2q}}{2}H}\;A\left(t\right)e^{-\frac{\beta_{2q}}{2}H}\;B\;\tilde{P}\;\right] \end{array}$$

where $C_{2q}$ and $R_{2q}$ are defined as usual from real and imaginary parts of $S_{2q}\left(t\right)=C_{2q}\left(t\right)+\hbar {\left(R_{2q}\right)}^{\prime \prime }\left(t\right)$ and correspond to fluctuations and response functions, respectively. Instead, the (Whiteman) correlation function $F_{2q}$ in the original space is

(49) $$F_{2q}\left(t\right)=\mathrm{Tr}\left({\left(\rho^{1/4q}A\left(t\right)\rho^{1/4q}B\right)}^{2q}\right)\;.$$

We remark that the Fourier transforms of the connected parts of $F_{2q}$, known as free cumulants, directly encode the energy shell correlations appearing in the eigenstate thermalization hypothesis [29,30].

The correlation functions defined in Equations (46)–(48) obey the FDT at a modified temperature $\beta_{2q}$ [31]. In the frequency domain, the FDT reads

(50) $$\begin{array}{cc} \;C_{2q}\left(\omega \right) & =\cosh\left(\beta_{2q}\hbar \omega /2\right)F_{2q}\left(\omega \right)\;, \end{array}$$

(51) $$\begin{array}{cc} \hbar {\left(R_{2q}\right)}^{\prime \prime }\left(\omega \right) & =\sinh\left(\beta_{2q}\hbar \omega /2\right)F_{2q}\left(\omega \right)\;, \end{array}$$

equivalent to the standard formulation $\hbar {\left(R_{2q}\right)}^{\prime \prime }\left(\omega \right)=\tanh\left(\beta_{2q}\hbar \omega /2\right)C_{2q}\left(\omega \right)$. We are interested in correlations in the time domain; hence, at the fluctuation–dissipation theorem formulated in the time domain, the *t*-FDT [9]. In particular, we will use the following relations:

(52) $$\begin{array}{cc} \;C_{2q}\left(t\right) & =\cos\left(\frac{\beta_{2q}\hbar }{2}\frac{d}{dt}\right)F_{2q}\left(t\right)\;, \end{array}$$

(53) $$\begin{array}{cc} \hbar {\left(R_{2q}\right)}^{\prime \prime }\left(t\right) & =\sin\left(\frac{\beta_{2q}\hbar }{2}\frac{d}{dt}\right)F_{2q}\left(t\right)\;. \end{array}$$

#### 4.2.2. The Bound

At times small but comparable with $t_{\mathrm{Ehr}}^{\left(2q\right)}$, the previous arguments showed that the 2q-OTOC are dominated by the regularized commutators [cf. Equations (38) and (39)]

$$C_{q}-C_{2q}\left(t\right)∼G_{2q}^{\left(\beta \right)}\left(t\right)\;,\;\;{\bar{C}}_{q}-F_{2q}\left(t\right)∼{\bar{G}}_{2q}^{\left(\beta \right)}\left(t\right)$$

when the behavior is exponential as $∼\exp[L_{2q}^{\left(\beta \right)}\left(t-t_{\mathrm{Ehr}}^{\left(2q\right)}\right)]$. The *t*-FDT in Equation (52) implies

(54) $$\frac{C_{q}-C_{2q}\left(t\right)}{{\bar{C}}_{q}-F_{2q}\left(t\right)}=\cos\left(\frac{\beta_{2q}\hbar L_{2q}^{\left(\beta \right)}}{2}\right)$$

The positivity of these coefficients—that follows from the fact that the 2q-th commutators are positive definite—requires that the GLE must be such that $\cos\left(\frac{\beta_{2q}\hbar }{2}L_{2q}^{\left(\beta \right)}\right)\geq 0$. We thus conclude

(55) $$\frac{L_{2q}^{\left(\beta \right)}}{2q}\leq \frac{\pi }{\beta \hbar }\;.$$

In the models where the Lyapunov depends on temperature [32,33,34], the cosine above in Equation (54) starts from zero at large temperature and is always in the first quadrant.

The bound on the 2q-th OTOC rate was previously derived by Tsuji et al. in Ref. [8], by taking Equations (38) and (39) as a working assumption. In Section 4.1 above, we justified it using the ordering of the Ehrenfest times $t_{\mathrm{Ehr}}^{\left(2q\right)}$.

### 4.3. Distribution Functions

The generalized Lyapunov exponents are the moments of a Lyapunov distribution function, as we have seen in the classical case and for the quantum GLE at an infinite temperature. In the case of finite β, the structure is more complex. This is due to the presence of q−dependent thermal matrices ρ in the definition of the regularized powers of commutators in Equations (29) and (30).

Nevertheless, one may define the Legendre transform of the thermal GLE as

(56) $$S\left(\lambda ,\beta \right)=\max_{q}\left(2\lambda q-L_{2q}^{\left(\beta \right)}\right)\;.$$

In analogy with the previous cases, we may interpret it as the Cramèr function of an associated large deviation function P(λ,β)∼exp(S(λ,β)t). As such, it shall obey similar properties as discussed above. In particular, the convexity of S(λ,β) and $L_{2q}^{\left(\beta \right)}$ corresponds with the ordering of the Ehrenfest times in Equation (6) assumed at the beginning. The latter is equivalent to the conditions that

(57) $$\frac{L_{2q}^{\left(\beta \right)}}{2q}\;\mathrm{increasing}\;\mathrm{function}\;\mathrm{of}\;q.$$

It is thus clear that the quantum bound (55) constrains the larger *q* that are related with the rare large deviations.

## 5. Discussion and Conclusions

In this work, we studied the quantum generalized Lyapunov exponents that quantify the large deviations of the spectrum of an appropriate operator. First, we discussed their convexity properties at infinite temperatures, which we exemplified on the kicked top. At finite temperatures, the quantum fluctuation–dissipation theorem (KMS) imposes a bound on their value, thus generalizing the celebrated bound to chaos to multipoint correlations. These bounds set a limit on the large deviations of chaotic properties.

A fascinating point is the interpretation of saturating the bound (7) at every *q*, which implies a form of mono-fractality:

(58) $$L_{2q}^{\left(\beta \right)}=\frac{\pi \hbar }{\beta }\;2q\;.$$

Classical examples of mono-fractal behavior, *i.e., models for which every trajectory has the same Lyapunov exponent*, are the backer map [35] and the free dynamics on the pseudosphere (the surface with constant negative curvature) [36]. What can we learn about the models that saturate the quantum bound (58)? A natural expectation is that the SYK model would lie in this class. In this case, it would be interesting to explore the meaning of such quantum mono-fractality in connection to the distinct properties of the model.

## Data Availability

Not applicable.
